# Forming new habits in the face of chronic cancer-related fatigue: An interpretative phenomenological study

**DOI:** 10.1007/s00520-021-06252-3

**Published:** 2021-05-06

**Authors:** Tom I. Bootsma, Melanie P. J. Schellekens, Rosalie A. M. van Woezik, Jenny Slatman, Marije L. van der Lee

**Affiliations:** 1 Scientific Research Department, Centre for Psycho-Oncology, Helen Dowling Institute, Professor Bronkhorstlaan 20, 3723 MB Bilthoven, the Netherlands; 2 Department of Culture Studies, School of Humanities and Digital Sciences, Tilburg University, Warandelaan 2, 5037 AB Tilburg, the Netherlands; 3 Department of Medical and Clinical Psychology, School of Social and Behavioral Sciences, Tilburg University, Warandelaan 2, 5037 AB Tilburg, the Netherlands

**Keywords:** Cancer, Chronic cancer-related fatigue, Habits, Survivorship, Qualitative research, Interpretative phenomenological analysis

## Abstract

**Purpose:**

The growing group of patients who suffer from chronic cancer-related fatigue (CCRF) after cancer have helpful and less helpful ways of responding to this long-lasting and disruptive problem. This qualitative study aimed to gain insight in essential elements of how patients respond to CCRF, with a focus on helpful responses to facilitate adaptation.

**Methods:**

We conducted semi-structured interviews with a purposive sample of 25 participants who experienced severe CCRF for at least 3 months. Participants were recruited via media, patient associations, meetings, and health professionals until data saturation was attained. We used a topic guide with open-ended questions about lived experiences. Interpretative phenomenological analysis (IPA) was used for analysis of the transcripts.

**Results:**

We identified five interrelated themes of how patients respond to CCRF: (1) discovering physical and emotional boundaries; (2) communicating support needs; (3) reorganizing and planning activities and rest; (4) letting go of one’s habitual identity; and (5) recognizing and accepting CCRF.

**Conclusion:**

This study highlights the development of new habits and positive beliefs in the face of CCRF and the importance of (social) support in this process. This experiential knowledge on helpful responses can be used to inform patients and their significant others and improve self-efficacy. Health professionals could use these insights to improve recognition of CCRF and personalize treatment.

**Supplementary Information:**

The online version contains supplementary material available at 10.1007/s00520-021-06252-3.

## Introduction

Approximately 25% of the cancer population experiences severe and disabling chronic cancer-related fatigue (CCRF) months to years after cancer treatment is finished [1–4]. Patients and their caregivers are often negatively impacted by the adverse physical, psychosocial, and economic consequences of experiencing CCRF [5]. For example, fatigue as a chronic illness can affect the ability of the patient to engage in paid employment [6] and caregiver burden is high [7, 8]. To date, various psychological and exercise interventions for CCRF exist and have been found effective in selected groups of cancer patients [9–11]. The variation in effectiveness of interventions for CCRF may also be down to a mixture of ineffective interventions or study designs that do not account for intra- and inter-individual variability in fatigue patterns. The question “what works best for whom?” needs to be addressed to support patients and therapists in the selection of the most effective intervention for alleviating CCRF. Further in-depth insight into the patient perspective on essential elements of responding to CCRF could help answer this question. Although the complex etiology is not fully understood, we expect that a variety of risk and protective factors can influence how people respond to CCRF over time [12, 13]. So far, the literature has mainly focused on unhelpful responses to fatigue (e.g., dysfunctional cognitions, dysregulation of activity, and negative social interactions) [8, 14, 15]. In order to improve treatment of CCRF, more insight in helpful responses is needed to target the complete spectrum of behavior during therapy.

A relevant interrelated framework of personal, behavioral, and social factors to gain insight into patients’ helpful responses to a chronic illnesses, such as CCRF, is the THRIVE model [16]. One of the key factors of this model is the forming of new habits and breaking with unhelpful habits [16–19]. From a psychological perspective, habits are defined as repetitive behaviors that are partially automatically executed over time [20]. The experience of cancer and its possible side effects during treatment has disrupted patients’ daily routines. The persistence of CCRF after treatment prevents patients from restarting their daily routines, meaning they cannot do simple (automated) tasks in daily life at the same time and in the same way as before cancer [5]. The formation of new habits is required in this situation to improve self-management behaviors, such as exercising [17, 19, 21]. The THRIVE model emphasizes the importance of positive psychological beliefs (e.g., acceptance of illness and self-efficacy) in order to adhere to these new habits in the long-term [22].

More insight into the formation of new habits and beliefs from a phenomenological perspective can inform us what conscious and unconscious processes are part of helpful responses in the face of CCRF. Therefore, we chose a phenomenological study design because this interpretative methodology can be applied to illuminate the first-person perspective of responding to CCRF. Responding to a chronic illness is expected to be a dynamic, complex, multifaceted, and ongoing process that is never fully completed [16, 23, 24]. The purpose of the present study is to gain insight in essential elements of how patients respond to CCRF, with a focus on helpful responding to facilitate adaptation.

## Methods

### Study population

Participants were deemed eligible when they met the following criteria: (1) adults at time of cancer diagnosis; (2) experienced severe cancer-related fatigue (score ≥ 35 on Checklist Individual Strength-fatigue severity subscale [25]) for at least 3 months after finishing curative cancer treatment (except hormonal treatment); (3) had no current or former severe psychiatric comorbidity (i.e., suicidal ideation, psychosis, or schizophrenia); and (4) were able to speak and read Dutch.

### Recruitment

All participants were recruited within two research projects in the Netherlands. The first project is the More Fit after Cancer trial (Fitter Na Kanker (FNK) Trial) that selected 22 participants after (partial) completion of one of the two online interventions for CCRF: physiotherapist-guided ambulant activity feedback (AAF) and psychologist-guided mindfulness-based cognitive therapy (eMBCT). In this study, nineteen participants agreed to participation in the interview study between December 2015 and March 2016. The second project is the REFINE project, in which 28 participants responded. Of the 28 responders, six participants were eligible and purposively sampled for participation in the interview study between August 2018 and January 2019.

In the FNK trial, participants were recruited via advertisements in newsletters of patient associations, relevant websites, regional newspapers, social media, and through oral presentations on various occasions for patients and/or caregivers. In the REFINE project, participants were recruited via patient websites or their health professionals. The health professional provided the participants with information about the interview study and referred interested participants to the researcher. Willing participants of both projects were then contacted by a researcher of one of both projects, and both verbal and written informed consent was obtained. We stopped recruitment when saturation was attained, i.e., when no new information emerged from the interviews of the purposive selected sample during analysis [24].

### Semi-structured interviews

The semi-structured topic guides consisted of open-ended questions based on literature and clinical experience and were pre-tested with a therapist with clinical expertise in treating patients with CCRF at the mental health institute for psycho-oncology. The first interviews with patients in both projects were seen as a pilot and evaluated as clear and concise. Therefore, no changes had to be made to the interview questions and these pilot interviews were included in the analysis. The topic guide of the FNK trial was focused on the evaluation of one of the two online interventions for CCRF (see Supplementary Materials Table S1). The topic guide of the REFINE project included experiences (descriptions, sensations, cognitions, patterns, attributions), consequences (daily life, body, self), actions (self, others, helping, and hindering factors), and other important factors related to CCRF (see Supplementary Materials Table S2). Interviews were face-to-face and held at the participants’ location of preference at either an institute for Psycho-Oncology, their home, or online with a video connection. Three researchers (two men, one woman), including the first author, conducted the interviews in both projects and had previous experience in psycho-oncology and/or qualitative research. The interviews were audio-recorded with a voice tracer and transcribed anonymously by one of the research assistants of the study projects. The duration of the interviews in the FNK trial was on average 40 min, and on average 64 min in the REFINE project. Member checks were utilized for the six additional interviews of the REFINE project by sending a summary of findings to participants to check that ways of responding to CCRF were correctly understood and to allow them the opportunity to respond. All participants agreed with the content of the summary.

### Data-analysis

The first and third authors independently coded the first five interviews of the FNK trial (MaxQDA software, version 18.2.0). Codes were discussed until consensus was reached. Because after five interviews, less and less variation between codes existed, the first author openly coded the other twenty interviews case-by-case in the same inductive way. In case of uncertainties regarding the codes, the first author discussed this with the third author to reach consensus. We used the six steps of interpretative phenomenological analysis (IPA) for the coding process [24]. The IPA is particularly useful to investigate the multidimensionality, dynamics, context, and subjectivity of CCRF [24]. The first step of coding according to IPA was reading and re-reading the transcripts to become familiar with the data. The second step was initial noting as the start of the open coding process. In the third step, emergent themes about responding to CCRF were developed within the interviews. A selection of six categories (i.e., metaphors, beliefs, comparisons, responses, helpful, and unhelpful responses) of the codebook were sorted in general maladaptive (e.g., move on, denial, and resistance) and adaptive (e.g., slow down, stop, or reduce activities) subcategories. The complete codebook was used for interpretative analysis of lived experiences with CCRF for publishing a separate paper. In the fourth step, we searched for connections across the emergent themes and developed a cross-table with two general patterns in the coping process: adapting (individual/social) and letting go (individual/social). In the fifth step, we checked for completeness with the summary of the individual interviews. In the last step, we organized two team discussions to identify superordinate themes that describe both helpful and unhelpful responses. Discrepancies about identified themes between team members were discussed until consensus was reached. This multidisciplinary team included all authors who have clinical and/or qualitative research expertise in psycho-oncology.

## Results

### Sample characteristics

In total, 25 patients suffering from CCRF participated. Nine participants (partially) completed the AAF intervention. Ten participants (partially) completed the eMBCT intervention. Two participants reported that they were currently treated by a physiotherapist. Eleven patients (44%) scored above the threshold (≥ 15) on the Hospital Anxiety and Depression Scale, suggesting clinical levels of distress [26–28]. Table 1 shows the characteristics of the participants.

**Table 1:** Participants’ characteristics (*N* = 25)

Characteristic	Value
Female/male gender (*n*)	16/9
Age at interview (years; mean ± SD, range)	52.64 ± 12.86 (21–80)
In a relationship (*n*)	23
Children at home (*n*)	11
Education level (*n*)^a^	
-Low	3
-Intermediate	13
-High	9
Currently employed (*n*)	
-Job	10
-(Partial) disabled	8
-No job/household	4
-Retired	2
-Scholar or student	1
Type of cancer(s) (*n*)^b^:	
-Breast^c^	10
-Hematological^d^	8
-Digestive system	5
-Head or neck	2
-Male genitalia	2
-Urinary tract	2
-Bone or soft tissue	1
-Brain and central nervous system	1
-Skin	1
-Gynecological	1
Treatment(s) (*n*)^b^:	
-Surgery	20
-Chemotherapy	18
-Radiotherapy	15
-Hormonal therapy	6
-Immunotherapy	2
-Bone marrow transplantation	1
Metastasis (*n*)	7
Comorbidities (*n*)^e^	12
Months since first diagnosis interview, (M ± SD, range)	56.88 ± 45.11 (11–169)
Months since last treatment interview, (M ± SD, range)	43.72 ± 35.96 (6–159)
Checklist Individual Strength (CIS)^f^, (M ± SD, range) Score subscale: fatigue severity (FS)	43.0 ± 5.02 (35–52)
Hospital Anxiety and Depression Scale (HADS)^f^ (M ± SD, range) score	13.84 ± 5.39 (4–23)
Duration of severe fatigue (*n*)	
-3–5 months	1
-6–12 months	6
-1–2 years	6
-2–5 years	7
- > 5 years	5
Prior professional support for cancer (*n*)^g^	18

aLow = primary/lower secondary education; intermediate = upper secondary education; high = higher vocational training/university.

bNumbers do not add up to 25 because multiple options are possible.

cOne participant had another type of breast cancer for the second time and was counted twice.

dOne participant had two different hematological diseases and was counted twice.

eComorbidities: diabetes, spine condition, pelvic disease, lung disease (asthma, bronchitis, or CARA), thyroid disease, liver disease, ulcerative colitis, graft versus host disease, sleep apnea, neuropathic sensations, vertigo, tinnitus, PTSS, neurological disease, migraine, and inflammatory bowel disease.

fBaseline data T0a FNK trial.

gProfessional support: revalidation, general practitioner, spiritual counsellor, social worker, psychologist, institute for mental health care, institute for psycho-oncology, psychosomatic exercise therapy, physiotherapy, and lifestyle program.

### Responses to CCRF

We identified five interrelated themes that characterize the dynamic and mutually reinforcing process of responding to CCRF: (1.) discovering physical and emotional boundaries; (2.) communicating support needs; (3.) reorganizing and planning activities and rest; (4.) letting go of one’s habitual identity; and (5.) recognizing and accepting CCRF. Table 2 shows patients’ quotes that support the five themes.

**Table 2:** Themes of responding to CCRF

1. Discovering physical and emotional boundaries
“*You will never reach your old level, so you have to keep in mind, maybe you will not reach that level ever again and be happy with what you can do. Yes, and in my case, I don’t want to exaggerate, but it all turned out fine. Thus, I can do just about anything what I could do before. I can play tennis again, I can go out for an evening, I can study and I can handle work. But I uh, I have noticed that I should be aware of to the fact that I will go very quickly beyond my boundaries*” [male, 31–40 years]
2. Communicating support needs
“*Yes, mainly my close friends and family notice this. And if I have a busy day at work and I’m unable to cook my meal without the risk of burning it than I can ask my sister if she would like to cook for me tonight*” [male, 31–40 years]
3. Reorganizing and planning activities and rest
“*Whenever I sit on a chair, I drink my tea in 5 min and then I move on. I’ve learned that’s not the way to do it. Instead, I get a puzzle, a Sudoku, then I solve the puzzle and remain seated for fifteen to twenty minutes*” [male, 61–70 years]
4. Letting go of one’s habitual identity
“*Yes, what is not helpful, is uh, doing sports [laughed] and yes, what shall I say, actually trying to pick up your old life. Of course, you return to life but not all activities you did before. That is not helpful actually. At least for me, that is counterproductive. I mean uh I can easily take my motorbike and so on but then I notice my concentration and I tried but it just doesn’t work and that is counterproductive so I leave it there. I tried, but. Just like work, I tried 3 h, but I couldn’t keep up*” [male, 41–50 years]
5. Recognizing and accepting CCRF
“*Well yes, specifically the fatigue comes up suddenly and it is actually not predictable when it will appear. Sometimes I am very busy and I do not feel it, and sometimes it’s a very quiet day and then all of a sudden, kaboom. But it is also a very strange paralyzing fatigue, fatigue that normally disappears when you sit down, but this doesn’t disappear. I still haven’t discovered, now after 6 years, how I can make it better. It comes and goes. And on the one hand I accept it, because it is a part of me, and I don’t know whether it is caused by the fact that I have had cancer or that it is caused by the chemo or the medications I still use. That I don’t know. But I know that I have it and I accept it and on the other hand I think, no, I just don’t accept it. Because I have to keep up in society, things are expected of me. And that makes it difficult to always accept it*” *[laughs a bit]* [female, 41–50 years]

#### Discovering physical and emotional boundaries

In the first period after experiencing CCRF, most patients used habits that were useful before their cancer diagnosis. For example, pushed themselves to do certain things in their daily life, tried to move on, and as a result, neglected their bodies and emotions. Moving on is characterized by speeding up activities and resisting CCRF. After a while, several patients stopped denying of the fact they suffered from CCRF and used different ways to tune into the emotions and sensations of their vulnerable bodies, afflicted by cancer and its treatment. For example, writing about their emotions, cognitions, and CCRF experiences helped discover change and progression. This kind of self-monitoring led to new insights into how their body felt and gave them a choice in how to protect their boundaries. The protection of their boundaries is a dynamic process of trial and error. Patients described that they needed to learn deciding on the right moment for relaxation to prevent exhaustion.

#### Communicating support needs

While discovering their physical and emotional boundaries, patients found it difficult to ask for help and tried to go on without support. However, most patients came to a point to accept they needed help and ask for the support of their close others. For example, patients openly communicated with their partner, friends, family, or colleagues about how they felt and what they could and could not do anymore. Most patients appreciated the support and empathic reactions they received from their social contacts. Some patients described how they tried to keep their CCRF silent when they met new people, because they were afraid of misunderstanding and negative reactions. When CCRF and the debilitating consequences for daily functioning persisted, most patients looked for more information on the internet, in books, or apps and/or asked for professional support from their medical oncologist, a psychologist, or physiotherapist.

#### Reorganizing and planning activities and rest

The experience of CCRF introduced a dysregulation in (social) activity, with patients being too active or too inactive. The time for rest was in all cases prolonged, for example, by sleeping more hours at night. Sometimes too much rest was taken, such as sleeping for hours during the day. As a result, patients did not experience an alleviation but an exacerbation of their feelings of CCRF. Becoming aware of their boundaries and communicating their support needs to others helped them to adapt their daily habits and search for more balance in activities and rest. Their activities were passively and actively clocked, structured, adjusted, or reduced, and alternated with time to rest. Patients reported examples of how they reorganized their daily activities: withdrawing from activities, taking time for themselves, going home earlier, and using ear plugs to avoid over-stimulation. Social activities lost their spontaneity and were planned. Patients prioritized the contact with close others and tended to stay at home instead of visiting someone. In cases of extreme fatigue, patients disengaged from their social life entirely and focused on resting. When exhaustion threatened their ability to do daily life activities, they found practical solutions. For example, some patients started using a disability parking card, cruise control in the car, an electric bike, or an elevator. The rush hours in traffic were avoided and a car was only used for short distances.

#### Letting go of one’s habitual identity

As patients learned more about their boundaries and adjusted their daily life accordingly, they were confronted with the fact the cancer and resulting CCRF had changed them. Some patients reported becoming more of an emotional person after cancer and preferring a different type of contact with others, with a focus on listening rather than talking and a need for in-depth conversations. Several (social) activities were not possible anymore. For example, some participants stopped working and had to let their significant others take over activities, such as taking care of children, driving the car, or cooking dinner. The letting go of old habits and becoming a less active person (in the evening or after activity) meant losing (part) of their old self.

#### Recognizing and accepting CCRF

Most patients experienced negative emotions, unhelpful thoughts, and beliefs that make it difficult to recognize and accept the unpredictable symptoms of CCRF. Some patients were able to let go of their negative emotions, unhelpful thoughts, and beliefs towards CCRF at certain moments throughout the day. If their social environment was accepting and understanding, it was easier to let go of their resistance against CCRF. The first step in this process was recognizing and acknowledging they suffered from CCRF. This awareness made it possible to accept that their situation was different from before cancer and they had to make the most of this “new normal.” The formation of new habits such as discovering their boundaries, adjusting their daily life activities, and communicating their support needs have contributed to the acceptance of CCRF.

### Interrelations of themes

The phenomenological approach showed that the identified themes are part of habit formation that starts at a pre-reflective level (i.e., prior to any conscious evaluation). One participant described the change in habits and adjustment to their new normal as an automatic process: “*Yes, unconsciously you devise things for yourself and yes that becomes routine and, in the meantime, you know, you don’t even realize that you have planned or managed things like that because it eventually has become normal. It actually has become your life…*” [male, 41–50 years]. The self-monitoring process of awareness of sensations and discovering physical and emotional boundaries is originated in the body and in relation to others by communicating support needs. These introspective and communicative ways of responding provide useful insights to reorganize one’s activities and rest and vice versa. The letting go of old and unhelpful habits and beliefs initiates an identity change, which creates room for new habits and beliefs that facilitate acknowledging and accepting CCRF.

## Discussion

This study aimed to better understand how patients respond to CCRF in helpful ways. The processes of forming new habits and positive beliefs and breaking with unhelpful habits and negative beliefs appeared essential for a helpful way of responding. Body awareness helped patients to discover their physical and emotional boundaries and seek support, which in turn facilitated time management between activities and rest. This change in habits created a change in identity, with new behavior and beliefs, which further aided patients to adhere to their new habits.

These findings are in line with other qualitative studies that found several comparable ways of responding to CCRF, such as support, activity management, identity change, and acceptance [29, 30]. However, these qualitative studies did not investigate the habit formation process with use of helpful and less helpful ways over time. The discovering of physical and emotional boundaries is not covered sufficiently by other studies. Another difference is that while other qualitative studies focused on differences of responding to CCRF between persons, our results suggest also the possibility of differences within persons of responding to CCRF throughout the day and from day to day.

These results build on previous findings that responding to CCRF is embodied [31] and partly an automatic, repetitive habitual behavior that requires minimal forethought [32]. The identified themes of responding to CCRF closely fit the three levels of habit formation that were reported by Wehrle, based on Husserl’s later works [33, 34]. These active and passive levels of habit formation represent both conscious and unconscious processes in relation to one’s previous experiences [33]. The first level of habit formation is defined as a style of experiencing based on a direct, unconscious reaction towards repeated individual experiences. The struggle against CCRF with unhelpful habits and beliefs, such as moving on and neglecting one’s body, are based on this primary unconscious reaction to CCRF and related to precancer experiences with fatigue. The second level of habit formation originates from the body and relates to previous embodied experiences (e.g., bodily memory) in active and passive ways. The themes “Discovering physical and emotional boundaries,” “Communicating support needs,” and “Planning and reorganizing activities and rest” are examples of the second bodily level of habit formation and indicate a learning process in relation to previous embodied experiences. For example, what the body experiences is central and related to previous experiences in the different self-monitoring processes patients use such as writing about experiences, clocking activities, and taking care of their bodies. At first, these processes are more active before becoming aware of their bodies in more passive ways. The third level of habit formation is based on personal and conscious reflection to change and adhere to new habits. The themes “Letting go of one’s habitual identity” and “Recognizing and accepting of CCRF” reflect this personal level of habit formation with development and adoption of new beliefs.

In the present study, we shed more light on what characterized the change of identity and how it plays a central role in responding to CCRF, by letting go of old habits and (social) activities and forming new ones. The changed habits of sleeping more, moving less, and changes in communication preferences make patients with CCRF sometimes unrecognizable for themselves and others, which has an impact on their identity [33]. This loss of self was reported by patients in several qualitative studies on CCRF [31, 35–37]. Informing and involving the social environment (e.g., partner) on what it means to experience and cope with CCRF can facilitate this change in habits [38].

### Clinical implications and future research

This study offers in-depth insight into the central role of the body, identity, and dynamics of helpful and unhelpful responses in the face of CCRF that can be useful for improving self-management and development of personalized treatment. It depends on the individual patient whether self-management is sufficient to form new habits and beliefs or whether additional treatment is needed, and which (combination of) treatment(s) is preferred and most effective in reducing CCRF. These insights into responses to CCRF can facilitate patients and therapists in making a shared decision about the preferred (combination of) treatment(s).

Because many patients suffer from multiple interrelated symptoms after anticancer treatment is completed, a transdiagnostic or holistic perspective is preferred to treat these patients. For example, Kuba investigated a group of hematological cancer patients (≥ 2.5 years post diagnosis) and found that acceptance of one’s present moment experiences is associated with lower levels of fatigue and subjective cognitive impairment [39]. Acceptance of CCRF is a central theme in our study and can be a potential target for interventions to deal with CCRF and other symptoms. Mindfulness-based interventions (MBIs) focus on enhancing bodily awareness by intentionally paying attention to present moment experiences, in an accepting, non-judgemental way [40, 41]. Besides face-to-face and group interventions for treating CCRF, effective web-based interventions (e.g., online activity coaching, cognitive behavioral therapy (CBT), and mindfulness-based cognitive therapy (MBCT)) might be a valuable alternative for patients [10, 42, 43]. Health professionals should advise patients about the different options and refer patients to the treatment of preference.

Future research on CCRF could benefit from innovative methodologies such as the experience sampling method and network analysis to investigate inter- and intra-individual differences in experiencing and responding to CCRF and find an answer to the question: what works best for whom? Further qualitative research for developing and evaluating interventions is recommended that include the caregivers’ perspective because responding to CCRF is a mutual process.

### Strengths and limitations

The IPA used in the present study goes beyond other existing qualitative studies on CCRF by exploring the central role of the body, interrelatedness, and importance of social context in the evaluation of helpful responses in a purposive selected sample.

Some limitations should be noted. First, patients’ experiences were evaluated retrospectively on different times after cancer treatment was finished. Second, although a diverse clinical sample of patients with CCRF and several comorbidities participated, we should be cautious to generalize the results to individual cancer patients with other cultural backgrounds and differing comorbidities. Third, similar to other qualitative studies on CCRF [31], the majority of participants had breast cancer which limits generalizability. At the same time, this is an adequate reflection of patients who seek psychological help for CCRF [44]. Fourth, although three different interviewers conducted the interviews, this impact is expected to be minimal, because of the self-reflection of interviewers and inductive participant-oriented analysis process of the rich data [24, 45].

## Conclusions

The present study highlights the development and adherence of new habits and beliefs in the face of CCRF and the importance of (social) support in this process. This new experiential knowledge on self-monitoring, support-seeking, and time-management habits and acceptance of CCRF can help inform patients and their significant others about self-management in the face of CCRF and improve self-efficacy. Health professionals could use these insights in clinical practice to improve timely recognition and personalize treatment for patients with CCRF.

## Supplementary Information

Below is the link to the electronic supplementary material.

Supplementary file1 (DOCX 20 KB)

## Data Availability

The datasets generated and/or analyzed in the current study are not available publicly as eligible patients were informed before start of the interviews that their data would be stored securely and confidentially.
