# Supplementary material for: Forming new habits in the face of chronic cancer-related fatigue: An interpretative phenomenological study
Source: Support Care Cancer. 2021 May 6;29(11):6651–9. doi: 10.1007/s00520-021-06252-3 (PMC8464573; doi:10.1007/s00520-021-06252-3)
Supplement: Supplementary file 1 — Supplementary file1 (DOCX 20 KB) [file 520_2021_6252_MOESM1_ESM.docx]

**Table S1 Topic guide More Fit After Cancer (FNK) trial**

| 1. **Before discussing your experience with the therapy: Can you tell me something about the symptoms or things you suffered from before you started in this study?**   **Prompt: fatigue, sleep, physical, anxiety, worrying, distress, work, social contacts, going out, family, household**   1. **Can you tell me something about your expectations before the start of the therapy?**   **Prompt: positive expectations, doubt**   1. **What was your experience with the therapy?**   **Prompt: positive and negative experiences**   1. **Can you tell me something about how you are doing now, after finishing treatment?**   **Prompt: fatigue, sleep, physical, anxiety, distress, work, social contacts, family, household**   1. **In what way do you think the therapy has been helpful?**   **Prompt: what and how?**   1. **Can you tell me something about how you experienced the contact with your therapist?**   **Prompt: How was the contact? Did you feel comfortable? Do you think following the therapy without a therapist would have been possible too?**   1. **Can you tell me something about how you experienced the exercises?**   **Prompt: Did the exercises help? With what and how? Was it pleasant or not? Where did you do the exercises? When did you do the exercises? What have you learned from the therapy? Do you still do the exercises? When? What exercises?**   1. **How did you experience receiving care via the computer?**   **Prompt: Did everything work, or were there times things did not work? How did you experience the online environment? Usability? How did you play the audio files? On what device? How did you read the booklet? Printed or on a device?**   1. **Do you have any other comments about the therapy not yet discussed?** 2. **How was it to participate in this study?** |
| --- |

**Table S2 Topic guide REFINE project**

| 1. **How would you describe your fatigue? How is it to be tired? What do you feel when you are tired? What do you think when you are tired?**   **Prompt: physical, mental, worrying, catastrophizing, cognitive and memory problems**   1. **I would like to know when you first suffered from this fatigue and how it has been going since then? Can you remember the moment the fatigue started? When did you first suffer from fatigue?**   **Prompt: duration, pattern (duration, frequency), changes over time, changes during the day,**  **comparison to pre-cancer fatigue**   1. **Maybe you have thoughts about what causes your fatigue? Why do you think you suffer from fatigue?**   **Prompt: triggers, sensory stimuli, cause, nutrition, changes in sleep, fear (for recurrence), worrying, distress, physical activity**   1. **How does fatigue influence your daily life and social environment?**   **Prompt: daily life, work, household, social life, partner/children/family**   1. **If you compare your life with fatigue with your life before the fatigue started, what has changed? How do you see yourself with fatigue?**   **Prompt: changes in view of the body, changes in looking at yourself, changes in identity, changes in how others see or approach you?**   1. **When the fatigue started, how did you deal with the fatigue? In what ways could you influence the fatigue?**   **Prompt: ways of dealing with fatigue, attributions, role of others (family, friends, acquaintances, others), to avoid/ be in situations?**   1. **What is helpful to you in dealing with fatigue?**   **Prompt: protecting or helping factors, attention, acceptation, activity**   1. **What is not helpful in dealing with fatigue?**   **Prompt: risk or unhelpful factors, go beyond boundaries**   1. **Do you have other experiences not yet discussed? What question about fatigue after cancer should I have asked to help understand it?**   **Prompt: other factors or questions**   1. **In order to improve treatment for patients with fatigue, I would like to know what is important in dealing with fatigue. What is your most important advice to yourself, others, professionals to better learn to deal with the fatigue?**   **Prompt: key topic, tips and tricks**   1. **How was it to participate in this study?** |
| --- |
